# Cross-Activation of Hemichannels/Gap Junctions and Immunoglobulin-Like Domains in Innate–Adaptive Immune Responses

**DOI:** 10.3389/fimmu.2022.882706

**Published:** 2022-07-15

**Authors:** Jiang-Hui Meng, Chang-Xu Chen, Mohammad R. Ahmadian, Hong Zan, Kai-Jun Luo, Jean X. Jiang

**Affiliations:** ^1^ School of Life Sciences, Yunnan University, Kunming, China; ^2^ Key Laboratory of the University in Yunnan Province for International Cooperation in Intercellular Communications and Regulations, Yunnan University, Kunming, China; ^3^ Institute of Biochemistry and Molecular Biology II, Medical Faculty and University Hospital Düsseldorf, Heinrich Heine University Düsseldorf, Düsseldorf, Germany; ^4^ Department of Microbiology, Immunology and Molecular Genetics, University of Texas Health Science Center, San Antonio, TX, United States; ^5^ Department of Biochemistry and Structural Biology, University of Texas Health Science Center, San Antonio, TX, United States

**Keywords:** connexin, pannexin, immunological synapse, T and B lymphocytes, cluster of differentiation antigens, phagocytosis, trogocytosis, transcytosis

## Abstract

Hemichannels (HCs)/gap junctions (GJs) and immunoglobulin (Ig)-like domain-containing proteins (IGLDCPs) are involved in the innate–adaptive immune response independently. Despite of available evidence demonstrating the importance of HCs/GJs and IGLDCPs in initiating, implementing, and terminating the entire immune response, our understanding of their mutual interactions in immunological function remains rudimentary. IGLDCPs include immune checkpoint molecules of the immunoglobulin family expressed in T and B lymphocytes, most of which are cluster of differentiation (CD) antigens. They also constitute the principal components of the immunological synapse (IS), which is formed on the cell surface, including the phagocytic synapse, T cell synapse, B cell synapse, and astrocytes–neuronal synapse. During the three stages of the immune response, namely innate immunity, innate–adaptive immunity, and adaptive immunity, HCs/GJs and IGLDCPs are cross-activated during the entire process. The present review summarizes the current understanding of HC-released immune signaling factors that influence IGLDCPs in regulating innate–adaptive immunity. ATP-induced “eat me” signals released by HCs, as well as CD31, CD47, and CD46 “don’t eat me” signaling molecules, trigger initiation of innate immunity, which serves to regulate phagocytosis. Additionally, HC-mediated trogocytosis promotes antigen presentation and amplification. Importantly, HC-mediated CD4^+^ T lymphocyte activation is critical in the transition of the innate immune response to adaptive immunity. HCs also mediate non-specific transcytosis of antibodies produced by mature B lymphocytes, for instance, IgA transcytosis in ovarian cancer cells, which triggers innate immunity. Further understanding of the interplay between HCs/GJs and IGLDCPs would aid in identifying therapeutic targets that regulate the HC–Ig-like domain immune response, thereby providing a viable treatment strategy for immunological diseases. The present review delineates the clinical immunology-related applications of HC–Ig-like domain cross-activation, which would greatly benefit medical professionals and immunological researchers alike. HCs/GJs and IGLDCPs mediate phagocytosis *via* ATP; “eat me and don’t eat me” signals trigger innate immunity; HC-mediated trogocytosis promotes antigen presentation and amplification in innate–adaptive immunity; HCs also mediate non-specific transcytosis of antibodies produced by mature B lymphocytes in adaptive immunity.

**Graphical Abstract:**
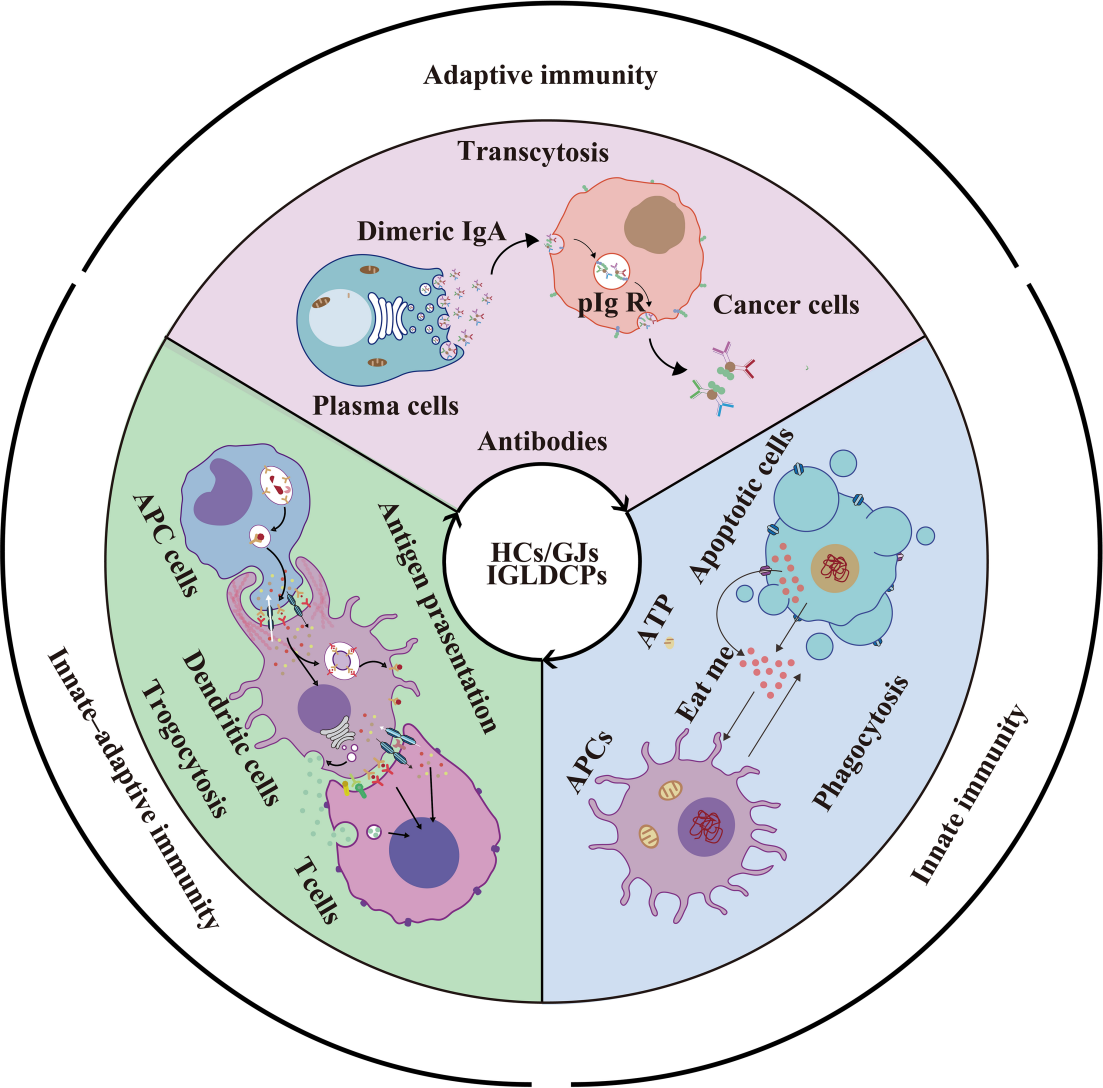
HCs/GJs and IGLDCPs mediate phagocytosis via ATP; “eat me and don’t eat me” signals trigger innate immunity; HC-mediated trogocytosis promotes antigen presentation and amplification in innate–adaptive immunity; HCs also mediate non-specific transcytosis of antibodies produced by mature B lymphocytes in adaptive immunity.

## Highlights

- Cx43 directly or indirectly interact with at least 20 IGLDCPs
- HCs/Panx release ATP to regulate APCs for initiating innate immunity
- Cx43-GJs between T cells and B cells activate adaptive immunity
- IgA induces APCs to transit adaptive immunity to innate immunity

## Introduction

Both types of immune responses, namely innate and adaptive, are linked to immune signal transduction. Hemichannel (HC)/gap junction (GJ)-mediated immune signal transduction in cells triggers an immune response. A similar immunological response is triggered by Ig-like domain-containing proteins (IGLDCPs). Both HCs/GJs and IGLDCPs localize on the immune cell surface to manipulate innate and adaptive immune responses. Previous studies have shown that the cross-activation of HCs/GJs and IGLDCPs is essential in mediating phagocyte migration, inflammation, and fever, among other successive stages of the innate immune response (1–6). In particular, in antigen-presenting cells (APCs), as well as T and B lymphocytes, HCs/GJs and IGLDCPs regulate the adaptive immune response (3, 7–11). However, numerous contentious issues persist, highlighting a potentially important goal: to elucidate the link between HCs/GJs and IGLDCPs in innate–adaptive immunity and provide available evidence on this potentially important topic.

Cell–cell communication during the immune responses confirms that HCs/GJs are closely involved in numerous cellular physiological processes. It is likely that antigen presentation, encompassing the T and B lymphocyte responses, involves in the regulation of cell migration and phagocytosis by pannexins (Panx) and GJ proteins, namely, connexins (Cxs) (12). Cxs form HCs on the cell surface; they can form both independent HCs and two HCs from two neighboring cells dock with each other to form intercellular gap junction channel. Conversely, Panxs form a structure, which is a single plasma membrane channel mediating extracellular communication. Cxs and Panxs are topologically similar with four transmembrane domains, two extracellular loops, one intracellular loop, and one N-terminal and one C-terminal. However, their potential interaction with IGLDCPs remains unclear.

The Ig-like domains are among the most widespread domains. Both sequence and structure of these domains can be found in diverse protein families. Proteins containing an Ig-like domain vary in their tissue distribution, amino acid composition, and biological function. IGLDCPs include immune checkpoint molecules of the immunoglobulin family expressed in T and B lymphocytes, most of which are cluster of differentiation (CD) antigens. The function of immune checkpoint modulators is to regulate immunological responses to infectious agents, foreign tissues, and cancerous cells; furthermore, they act to balance the immune response through either enhancement or inhibition (13–16). However, there are relatively few studies on the regulation of HCs/GJs by IGLDCPs

Although there is limited understanding of the interactive mechanisms between HCs/GJs and IGLDCPs, we have attempted to present a rational and balanced evaluation to bridge this gap. In the present review, several important questions have been raised on the seminal findings. HCs/GJs, which mediate intracellular and extracellular communication, are involved in immune response regulation. The following pertinent questions arise. Do HCs/GJs directly interact with IGLDCPs, including immune checkpoint molecules such as CD antigens, to regulate innate–adaptive immunity? Do HCs/GJs regulate IGLDCPs in T and B lymphocytes? Although both HCs/GJs and IGLDCPs regulate phagocytosis, what is the physical and function relationship between them? How do HCs/GJs and IGLDCPs trigger trogocytosis and transcytosis?

## Mutual Cross-Activation of HCs and IGLDCPs

### Cxs and Panx1 on the Immunological Cell Surface

**Figure 1:**
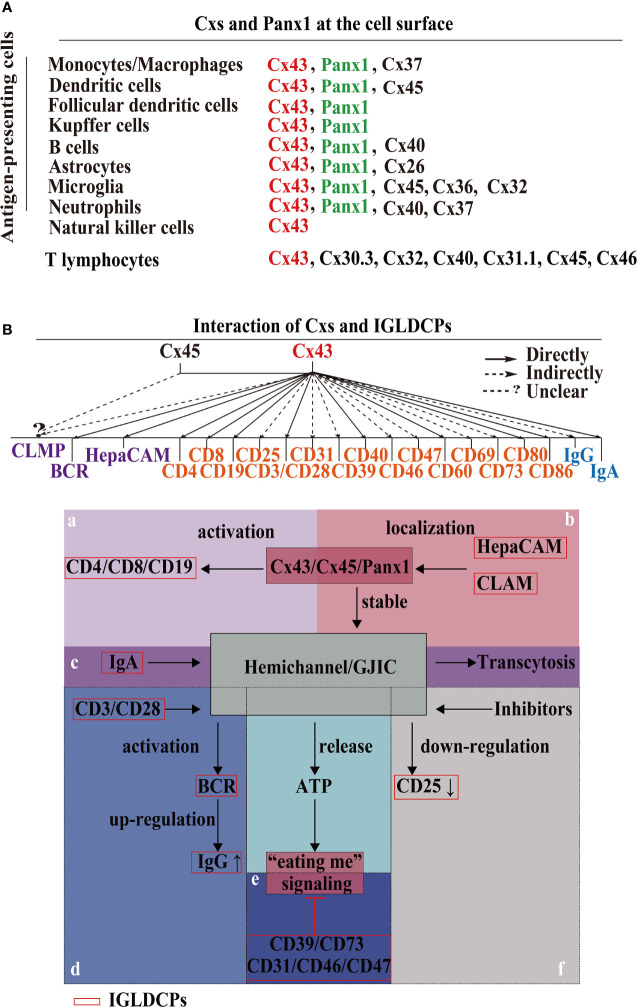
Cxs and Panx1 on the immunological cell surface and their mutual interaction with IGLDCPs. **(A)** Cxs and Panx1 localize at the cell surface. Cxs and Panx1 HCs have been identified in nine types of APCs and T lymphocytes, primarily for the signaling role of HCs with IGLDCPs in the innate–adaptive immune response (17, 18). **(B)** The mutual interaction of HCs and IGLDCPs. Cx43, which interacts with at least 20 IGLDCPs: a The activation of CD4/CD8/CD19 requires Cx43/HC (19–21). b HepaCAM and CLAM facilitates Cx43 membrane localization and GJIC establishment (22–24). c GJIC mediates the transcytosis of IgA in CD19^+^ B cells (21). d CD3/CD28/CD40 activate BCR signaling and upregulate IgG expression by Cx43/HC opening (25). e The “eating me” signaling pathway is inhibited by CD39/CD73/CD31/CD46/CD47 (14, 26). f CD25/69 are downregulated by the inhibition of Cx43/HC (3). Cxs, connexins; Panx, pannexin; IGLDCP, Ig-like domain-containing protein; HC, hemichannel; APC, antigen-presenting cell.

Cxs are localized on the cell membrane of at least nine subtypes of APCs, namely, monocytes, macrophages, dendritic cells (DCs), including follicular dendritic cells (FDCs), Kupffer cells, B cells, astrocytes, microglia, neutrophils, and natural killer (NK) cells. Panx1 is also found on the eight subtypes of APCs (**Figure 1A**). As shown in **Figure 1A**, Cx43 is expressed in the aforementioned nine APC subtypes. Moreover, Cx37 is expressed on macrophages and neutrophils; Cx45 is present on the surface of DCs and microglial cells; Cx40 on the membrane of B cells and neutrophil cells; and Cx26 on astrocytes. In addition to those listed above, Cx36 and Cx32 are expressed on the microglial cell surface. Seven Cxs, namely, Cx30.3, Cx31.1, Cx32, Cx40, Cx43, Cx45, and Cx46, are present in T cells (4, 17, 18, 27–31). Unexpectedly, 8 of the 21 Cxs in the human gap junction protein family serve as components of synapses or participate in them. There are probably more Cx subtypes that remain to be identified in future research, as most previous studies focused on immune checkpoint molecules without conclusively evaluating Cxs in innate–adaptive immunity. Therefore, elucidating the interaction between HCs and IGLDCPs will offer mechanistic insights into the innate–adaptive immune response. In the following section, we have further detailed the mutual interaction between Cxs and IGLDCPs.

### Mutual Interaction of Cxs and IGLDCPs

Cx43 interacts extensively with at least 20 IGLDCPS, either directly or indirectly, namely, CLMP, BCR, HepaCAM, CD4, CD8, CD19, CD25, CD3/CD28, CD31, CD39, CD40, CD46, CD47, CD60, CD69, CD73, CD80, CD86, IgG, and IgA (**Figure 1B**). Cx43 regulates T lymphocytes and DCs *via* IGLDCPs. Cx43/Cx40 maintains lymphocyte homeostasis and cytokine production, such as in the case of Cx43 HC inhibition, which suppresses IL-2 and IL-6 mRNA expression (32). Furthermore, the use of mimic peptides as an inhibitor of Cx43 HCs downregulated CD69 and CD25 activation in T cells, and led to IFN-γ by release by DC-stimulated NK cells (3). Similarly, CD3/CD28 induced ATP release by γδT cells, aided by HCs, resulting in cell activation (33, 34). Furthermore, Cx43 HCs in the plasma membrane of CD4^+^ T lymphocytes establish gap junction intracellular communication (GJIC) with macrophages to synthesize and secrete Igs and cytokines in immune regulation (19). Similarly, in DC–DC interaction, CD80, CD 86, and MHC class II are expressed (33, 35). Directly, Cx43 activates spleen cells and facilitates IgG production. Targeting *Cx43* is a potential strategy to treat diseases associated with the antibody response (14). Cx43 regulates B lymphocyte activation directly, through BCR signaling, which involves migration and motility (7, 36).

The IGLDCPs regulate Cx43 HCs/GJs. The Ig-like domain in hepatocyte cell adhesion molecule (hepaCAM) stabilizes the Cx43 HCs on the cell surface. The hepaCAM gene was first described in hepatocellular carcinoma and was also discovered in the central nervous system (CNS); it is named GlialCAM, based on the site of its identification (37). HepaCAM is reportedly a member of the immunoglobulin superfamily (IgSF); it consists of an extracellular domain with two Ig loops, a transmembrane region, and a cytoplasmic tail, and functions in conjunction with Cx43 (38). In previous studies in U373 MG glioblastoma cells studies, it was found that hepaCAM expression redistributes Cx43, especially to the site of cell–cell contact, where co-localization of the two molecules is detected (38). Furthermore, altering the Ig-like domain of hepaCAM, especially the first extracellular IGLDCP reduces the co-localization of intercellular Cx43. Cx43 is shuttled back to the cytoplasm from the cell membrane, consequently decreasing its membrane-bound expression. In summary, the presence of IGLDCPs stabilizes Cx43 expression and promotes transport of a protein localized in the cytoplasm to the cell surface (22, 23). Additionally, CAR-like membrane protein (CLMP) regulates Cx43 and Cx45, and the absence of CLMP causes functional obstruction due to a lack of GJIC (24). Cxs and IGLDCPs are co-localized and interact at the immunological synapses (ISs). An increased intracellular Ca^2+^ level, which induces T cell activation and signal amplification, is facilitated by IS Cx43 HCs. Therefore, the formation of ISs is an important function structure, which allows us to understand how HCs/GJs and IGLDCP collaboratively modulate the precise roles in innate–adaptive immune responses.

## HCs/GJs and IGLDCPs Interact to Form the Synapse

**Figure 2:**
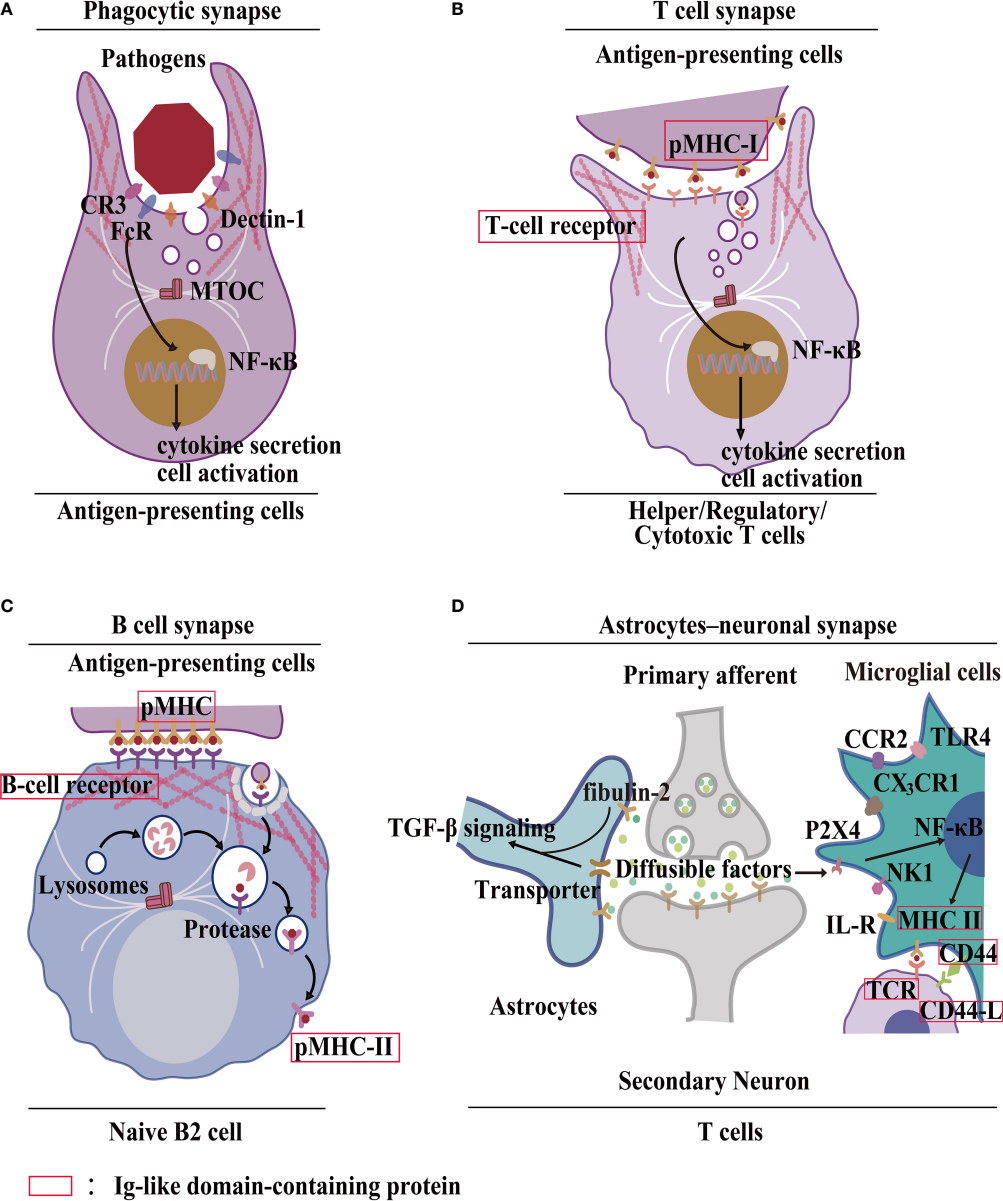
Formation of the IS by HC–IGLDCP interaction. **(A)** A phagocytic synapse formed by APCs. Phagocytes possess specific molecules on the synaptic surface that trigger phagocytosis. For example, recognition of Fcy receptor (FcR) sites, CR3 envelope site, Dectin-1 receptor trigger site (41). MTOC: Center for Microtubule Organization. **(B)** T-cell immunological synapse. A specific cellular contact between T cells and APCs. Major histocompatibility complex (pMHC-I) molecules on the surface of APCs bind to the T-cell receptor (TCR) and deliver the antigen, leading to the polarization of APCs by T cells and the coordinated recombination of various components of T cells, including signaling molecules and adhesion molecules, actin and microtubule cytoskeleton (41). **(C)** B-cell immunological synapse. A specific cellular contact between B cells and APCs. The pMHC-I molecules are phagocytosed in a clathrin-dependent manner. The antigens are transported to lysosomal vesicles for digestion, and the resulting peptides are loaded onto MHC-II molecules and transported back to the cell surface for presentation to T cells (42). **(D)** Neuronal Synapse. It consists of neurons, astrocytes, microglia, and T cells. Astrocyte-derived extracellular vesicles promote synapse formation through fibrin 2-mediated TGF-β signaling. Microglia MHC-II protein, CD40, and other stimulating molecules recruit T cells to deliver antigens. Different receptors bind to different ligands (43, 44). HC, hemichannel; IGLDCP, Ig-like domain-containing protein; Cx, connexin; Panx, pannexin; APC, antigen-presenting cell.

Importantly, HCs/GJs are complex signaling components of the ISs (39, 40)—phagocytic synapse, T cell synapse, B cell synapse and astrocyte–neuronal synapses. First, HCs are involved in phagocytic synapse formation between APCs and pathogens (41) (**Figure 2A**). Cx HC-linked “pathologic pores” are involved in spreading injury and perpetuating chronic disease. Opening HCs are involved in spinal cord injury progression and the spread of cellular edema. They also control important aspects of the innate–adaptive immune response, particularly under chronic disease conditions, as well as the initiation and perpetuation of the inflammasome pathway in astrocytes (45). It has been reported that Cx43 also regulates FDC development (46), implying Cx43 may form a phagocytic synapse and perform important functions, which warrant further research. Second, the T cell synapse contains GJs. GJIC established by Cx43 is an important functional component of the T cell synapse (41) (**Figure 2B**); it also activates T cells by sustaining the communication between T cells and APCs (11, 19, 47–49). Furthermore, in melanoma cancer cells, Cx43 GJIC plaques localized at the IS are required for augmenting granzyme B activity, to enable cytotoxic T lymphocytes (CTLs) to kill B16F19 melanoma cells (50). It has been reported that Cx43 GJIC between DCs and also activated T cells (51). These findings confirmed that Cx43 plays a vital role in the T cell synapse. Additionally, Cx43-forming HCs/GJs activate the T cell IS (25). Third, the B cell synapse is formed between B cells and APCs (42) (**Figure 2C**). However, compared with phagocytosis and T cell synapses, data on B cell synapse are rather limited. These findings demonstrated that the HCs/GJs are an intrinsic part of the ISs and are essential to mediate IS intracellular communication in regulating the delivery of immune factors.

The astrocyte–neuronal synapse is established between neurons and astrocytes, which release diffusible factors to activate microglia *via* NF-κB signaling (41, 52–54). Astrocytes interact with neuronal synapses to establish astrocyte–neuronal communication (55). Research has shown that astrocyte-derived extracellular vesicles promote synaptic formation through fibrin 2-mediated TGF-β signaling. Consequently, microglia MHC-II protein, CD44, and other molecules recruit T cells for effective antigen delivery (43, 44) (**Figure 2D**). Investigating the interaction between HCs/GJs and IGLDCPs in the astrocyte–neuronal synapse presents a worthwhile research opportunity. Interactions of HCs/GJs and IGLDCPs with IS provide direct evidence suggesting that both may play an important role in immune responses.

## Cx/Panx and IGLDCPs Display Dual Functions in Innate Immunity

### ATP “Eat Me” Signaling, as Well as CD31, CD46, CD47 “Don’t Eat Me” Signal Molecules, Triggers Phagocytosis

**Figure 3:**
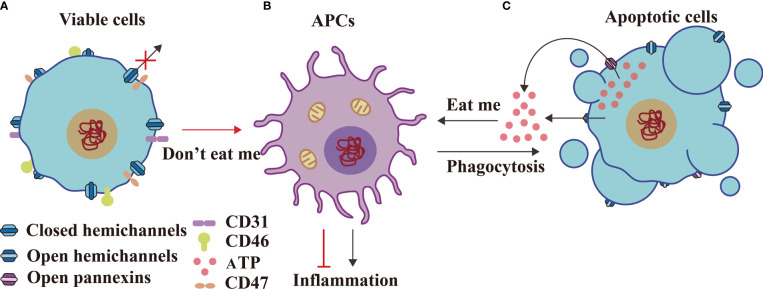
HCs and IGLDCPs display dual functions in innate immunity. Viable cells form closed HCs **(A)** with APCs **(B)** transmitting “don’t eat me” signals, including CD31, CD47, and CD46, to suppress inflammation. In contrast, apoptotic cells **(C)** send an “eat me” signal to APCs by opening the HCs and releasing ATP, which engulfs apoptotic cells and causes inflammation (56). HC, hemichannel; IGLDCP, Ig-like domain-containing protein; APC, antigen-presenting cell.

Panx1 releases ATP from apoptotic cells to trigger an “eat me” signal (56) (**Figures 3A–C**). Key phagocytic inducers, ATP and UTP, have been confirmed to recruit apoptotic cells *in vitro* and *in vivo*. In contrast, “don’t eat-me” signals comprise CD31, CD46, and CD47 expression. These signals on healthy viable cells, which are capable of phosphatidylyserine (PtdSer) exposure under physiological conditions, may positively inhibit phagocytic uptake (26). These findings elucidate the mechanism governing HC–IGLDCP interaction in phagocytosis.

In the macrophage cell line J774, Cx43 RNAi showed impaired phagocytosis of the polystyrene-covered beads, and sheep erythrocytes opsonized by IgG (47); in contrast, in sheep erythrocytes with heterozygously or homozygously deleted *Cx43*, no changes were observed in phagocytosis (12, 57). Recently, Dosch *et al.* assessed Cx43 function in phagocytosis using *Cx43* deletion and inhibition. It was determined that the inhibition of autocrine communication of Cx43-dependent ATP in macrophages improved sepsis outcome (33, 48, 49). Therefore, full expression of intact Cx43 is essential in regulating of the immune response through the directionality and rate of DC migration (58). Different cytokines regulate intercellular communication, facilitated by HCs/GJs in APCs, to execute purinergic signaling (3). This presents an interesting research opportunity for further investigation of Cx43-macrophage-phagocytosis. Apoptotic cells attract phagocytes by releasing chemotactic factors known as “find-me” signals (26).

### Cx/Panx and IGLDCPs Regulate the Inflammatory Response

**Figure 4:**
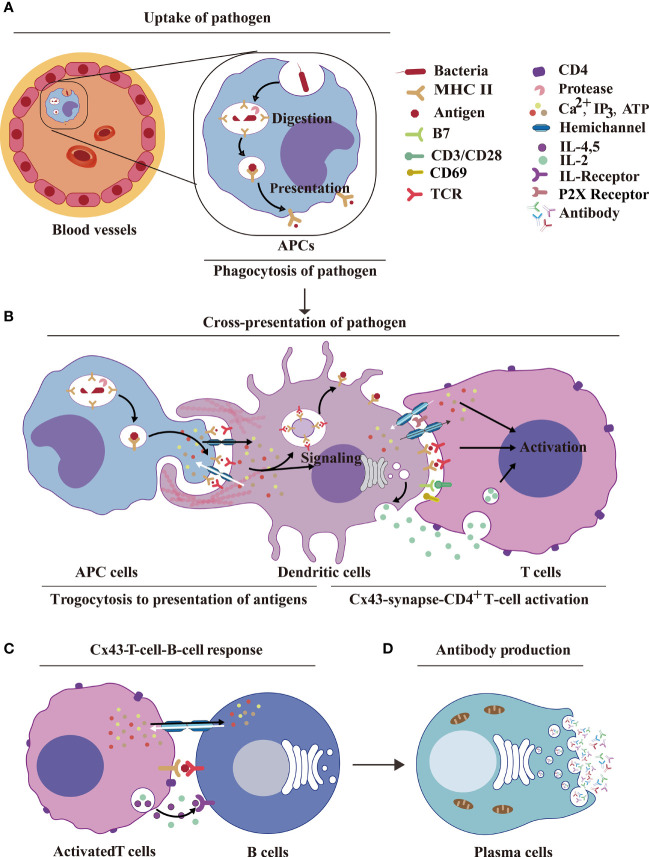
HCs and IGLDCPs activate innate–adaptive immunity. **(A)** Uptake of pathogens by APCs. The pathogenic antigens are phagocytosed by APCs and digested by proteases to form peptides, which are then transmitted by MHC molecules to the cell surface by phagocytosis. **(B)** Dendritic cell Cx43s are dependent on trogocytosis for antigen delivery to T cells. The antigen is processed by APCs and binds to MHC receptors on the APC membrane. The HCs and IGLDCPs in innate–adaptive immunity recognize and remember specific pathogens to trigger immunity. The former facilitates ATP release and autocrine feedback mechanisms that control Ca^2+^ entry. GJs between monocytes and CD8^+^ DCs transfer antigens *via* Cx43-synapse-CD4^+^ T cells (59, 60). **(C)** Cx43-T-cell–B-cell response. The activated T cells facilitate the opening of HCs, which liberally release ATP into B cells. This results in the simultaneous release of IL-2, IL-4, and IL-5, which act on the IL-R receptor and further stimulate B cells to produce antibodies. **(D)** Antibody production. Activated B cells form plasma cells, which produce antibodies (61). HC, hemichannel; IGLDCP, Ig-like domain-containing protein; APC, antigen-presenting cell; MHC, major histocompatibility complex; DC, dendritic cell.

Cumulative evidence shows that ATP triggers the inflammatory response. Cx HCs serve as a major pathway for the release of cytoplasmic ATP into the extracellular space. In granulocytes, Cxs enhance the inflammatory responses and promote cellular activation (33) (**Figure 4B**). For example, ATP released by Panx1 promotes the opening of Cx43 HCs (62) and is also involved in the innate immune response and inflammation (26, 63–70). In contrast, the blockage of Cx43 isoform HCs alleviates inflammation and enhances healing (2). In other inflammatory pathologies, Cx43 expression regulates monocyte–endothelial adhesion, with criteria for baseline adhesion set by Cx43-expressing monocytes (71). Similarly, elevated macrophage Cx43 HC activation and *Panx1* expression inhibit pathogenesis (1, 49). Cx43 GJs transfer hypoxia-induced miR-192-5p, allowing cancer cells to acquire immune-resistant phenotypes (25). During inflammation in response to spinal cord injury, a decrease in the expression of Cx43 proteins shortens animal recovery time (33). ATP release has been inhibited using several Cx43 mimic peptides, thereby influencing the inflammatory process (72). Therefore, ATP integrated with the HC function promotes inflammation.

Open Cxs HCs in macrophages facilitate an effective immune response. GJs and HCs help spread toxicity into neighboring areas to augment viral/bacterial replication, and promote the spread of the inflammatory response by infectious agents, such as HIV (33),. Ig-like domains presenting T cell immunoglobulin molecules regulate inflammation and immune responses (16, 73). Single immunoglobulin IL-1R-related molecule (SIGIRR) is a specific inhibitor of IL-1R and toll-like receptor signals (74, 75).

In summary, Cxs HCs and Panx1 release ATP, which serves as an “eat me” signal; conversely, CD31, CD47, and CD46 function as “don’t eat-me” signals, which regulate phagocytosis in innate immunity.

## Cx43 and IGLDCPs Activate Innate–Adaptive Immunity

### Cx43-Dependent Trogocytosis of Macrophages and Dendritic Cells in Antigen Presentation

The interaction between innate and adaptive immune response is defined as innate–adaptive immunity, which is important for antigen presentation. Cx43 contributes to trogocytosis (**Figures 4A, B**). The mechanism of innate control of adaptive immune responses involves multiple signaling pathways (16). We focused on how macrophages detect pathogens or injured cells. Trogocytosis is a process whereby lymphocytes extract surface molecules of APCs and express them on their own membranes (59, 60) (**Figure 4B**). However, the role of HCs and IGLDCPs in trogocytosis has not been examined adequately. *Cx43*-deleted macrophages are more proficient in T cell priming, implying an increased accumulation of antigens as these macrophages cannot transfer them to neighboring DCs, resulting in efficient presentation (57, 76–80). These findings delineate a potential mechanism by which HCs and IGLDCPs regulate antigen presentation.

GJs also have a pivotal function in DC activation and the amplification of antigen presentation, such as antigen transport, dendritic activation, and antigen cross-presentation (10, 33, 81–83). GJs-mediated antigen transfer between monocytes and CD8^+^DCs may serve as a simple and efficacious immunotherapy strategy for cancers, such as in the case of undifferentiated monocytes loaded with tumor antigen (20). Molecules containing Ig-like domains, such as pMHC-I and II, are involved in Cx43-dependent trogocytosis on the surface of acceptor cells (78). This is a valuable research direction to explore the underlying mechanism by which Cxs and IGLDCPs regulate antigen presentation *via* trogocytosis.

### Cx43/Panx-Mediated Activation of CD4^+^ T Lymphocytes

In addition to the roles of Cx43 in regulating macrophages and dendritic cells. HCs also mediate CD4^+^ T lymphocyte activation is critical in the transition of the innate immune response to adaptive immunity (**Figure 4C**). In a previous study, it was found that Cx43 in the IS delivers microRNAs from hypoxic melanoma cells to CTLs (25). Therefore, Cx43 stimulates T lymphocytes by the delivery of immune factors. Cx43 is involved in the formation of GJs in CD4^+^ T lymphocytes, Th0, Th1, and Th2, and macrophages; this pathway was found to be especially prominent in Cx43-Th1–macrophage interaction (19). This, in turn, suggests the potential capability of HCs in controlling IGLDCP activation. Cx43-GJs at the IS between DCs and CD4^+^ T cells promote T cell activation during antigen presentation (11), whereas the inhibition of GJs hindered DC-mediated T cell activation, reflected by lower T cell proliferation, CD69 expression, and IL-2 secretion.

Interestingly, in the absence of DCs, Cx43 GJ blockers did not affect the activation of CD4^+^ T cells triggered by anti-CD3/anti-CD28. Therefore, it was inferred that suppression of Cx43 inhibits Cx43 GJ assembly between DCs and T cells, resulting in T cell inactivation (84). In the DC–T cell IS, the blocking of Cx43 HCs/GJs (on either DCs or T cells) inhibited IFN-γ secretion and decreased the intracellular Ca^2+^ concentration, upon interaction of T cells with antigen-loaded DCs. These results strongly suggested that Cx43 HCs act in signaling amplification and T cell activation, by either releasing ATP or taking up of inositol triphosphate (IP_3_) from DCs (25).

Cx43-GJs amplify antigens to activate T lymphocytes *via* the antigen cross-presentation pathway. In the immune synapse, Panx1, which releases ATP, controls Ca^2+^ entry to activate T cells; this happens by stimulating autocrine/paracrine receptors, such as P2X1 and P2X4 (85). Cx43-GJs between monocytes and CD8^+^ DCs transfer antigens (20), whereas Cx43 HCs activate CD4^+^ T cells (86). Together, Cx43-dependent trogocytosis of macrophages and dendritic cells promote antigen uptake, transfer, and presentation to activate innate–adaptive immunity. The GJ protein Cx43 induces B lymphocytes (8) to produce antibodies in plasma cells (61) (**Figure 4D**).

## Cxs and IGLDCPs Mediate Adaptive Immunity and IgA Transits Adaptive Immunity to Innate Immunity

### Cx43-CD39/CD73-Treg-Mediated Immunosuppression

**Figure 5:**
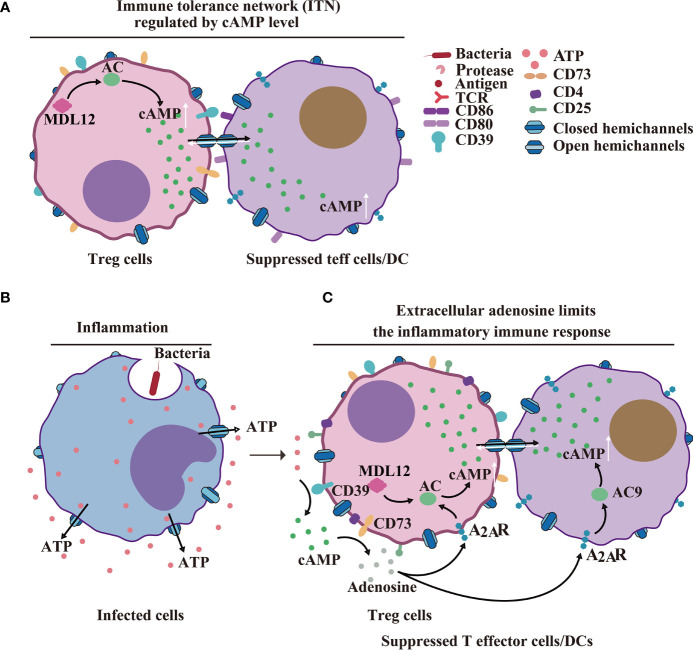
HCs/GJs and IGLDCPs mediate adaptive immunity. **(A)** Cx43-cAMP cell-mediated immune response. Regulatory T cell-mediated inhibition of naturally occurring Treg cells and conventional T cells delivers cAMP to responder T cells *via* GJs to inhibit T-cell proliferation and IL-22 synthesis (89). **(B)** HCs in infected inflammatory cells release ATP. **(C)** Extracellular adenosine limits the extent of the inflammatory immune response. Activated CD4 ^+^ T cells. The activated CD39 ^+^ cells release cAMP *via* paracrine signaling, to suppress T effector (Teff) cell and dendritic cell (DC) functions (89). HC, hemichannel; IGLDCP, Ig-like domain-containing protein.

In cellular suppression mechanisms, naturally occurring Treg cells and helper T cells communicate *via* GJs to deliver cAMP to responder T cells, thereby inhibiting T cell proliferation and IL-2 synthesis (87–89) (**Figures 5A–C**). In a recent review, it has been reported how the cross-talk between Cxs and cAMP regulates cell-cycle progression, particularly in cancer cell populations (90). Furthermore, Cx43 expressed by thymic Treg cell progenitors supports Treg cell development. Conversely, *Cx43* deletion decreased the number of functional Tregs and increased non-functional CD4^+^CD25^+^GITR^+^FOXp3^-^ T cells, which are incapable of producing inflammatory cytokines and inhibiting cancer cell progression (91). In human Treg cells, it has been shown that CD4-mediated activation involves elevation in the intracellular cAMP concentration. In contrast, the decrease in the cAMP level, caused by the application of adenylate cyclase (AC) inhibitor MDL12, resulted in the proliferation of Treg cells, *in vitro* and *in vivo* (87, 89) (**Figure 5B**). Consequently, it is inferred that Cx43 HCs may release cAMP; however, this needs to be studied further.

Cx43-GJs accumulate at the cytotoxic IS, enabling CTL-mediated melanoma cell killing (50). Additionally, Cx43 regulates the proliferation of CD4^+^CD25^+^ T lymphocytes and production of cytokines (92). Cx43-GJs regulate CD4^+^CD25^+^ Treg lymphocyte activation and inflammatory cytokine (IL-2 and IL-6) production in hypertensive inflammation in the spleen of rats (32, 92).

### CD19^+^ B Cell IgA Transcytosis Transits Adaptive Immunity to Innate Immunity

Recently, it was determined that tumor antigen-specific and tumor antigen-independent IgA transcytosis and antigen regulate ovarian cancer immunity. Tumor B cell-derived IgA binds to the polymeric immunoglobulin IgA receptors (pIgR) on ovarian cancer cells and reprograms myeloid cells against extracellular oncogenic drivers, such as EGFR and KRAS, which causes cell death. In particular, innate immunity triggered by antigen-independent IgA transcytosis is a novel strategy. IgA transcytosis through malignant epithelial cells causes tumor cells to encounter cytotoxic T cells, thereby hampering malignant progression; furthermore, the associated transcription changes result in suppression of the RAS pathway (21). In the ovarian cancer immunological response, IgA, B cells, and atypical B cells are observed (93). Transcytosis is a process in which molecules cross cellular barriers, which includes pinocytosis, endocytosis, and trafficking of vesicles to the opposite membrane (94).

In summary, Cx43-CD39/CD73-Treg-immunosuppression mediates adaptive immunity, specifically, IgA transcytosis, with tumor antigen-dependent and -independent mechanisms. It also regulates the establishment of immunity in ovarian cancer.

## Concluding Remarks and Future Perspectives

**Figure 6:**
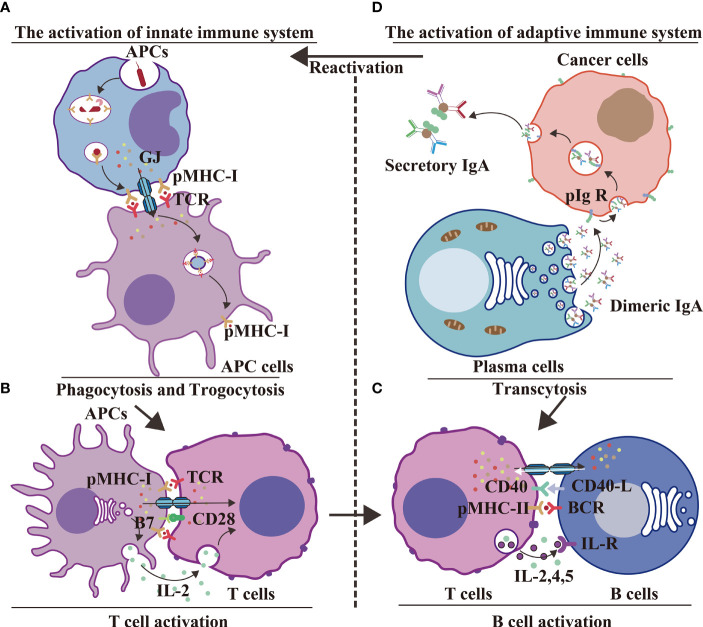
GJs and IGLDCPs regulate phagocytosis, trogocytosis, and transcytosis during innate–adaptive immunity. **(A)** Innate immunity – antigen production. APCs receive pathogens and form epitopes on the membrane surface through digestion and processing of antigens. Interaction of GJs with IGLDCPs triggers phagocytosis and trogocytosis, thereby resulting in T-cell activation (14, 50). **(B)** Innate immunity – T-cell activation. Interaction of GJs with IGLDCPs: the former delivers Ca^2+^ and ATP to T cells, whereas the latter, in contact with CD28 and facilitated by paracrine IL-2 signal transmission, activates T cells. **(C)** Adaptive immunity – B-cell activation. After CD4^+^ T-cell activation, pMHC-II establishes contact with the B-cell receptor (BCR). The resultant release of IL-2, IL-4, and IL-5 leads to B-cell activation. The adaptive immune response is jointly mediated by GJs and IGLDCPs (95). **(D)** Adaptive immunity – Antibodies affect pathogens. GJs activate the adaptive immune response to generate antibodies **(D)**. Conversely, IgA can induce APCs to activate the innate immune response *via* transcytosis **(A)** IgA can also promote B-cell activation **(C)** (96). HC, hemichannel; IGLDCP, Ig-like domain-containing protein; APC, antigen-presenting cell; GJ, gap junction.

In conclusion, GJs between two APCs interact with pMHC-1 of phagocytic APCs and TCR of trogocytic APCs to execute antigen delivery (14, 50) during innate immunity (**Figure 6A**). GJs interact with pMHC-I, B7 from APCs and TCR, CD28 from T cells to facilitate Ca^2+^-mediated T cell activation (**Figure 6B**); GJs interact with CD40, pMHC-II from activated T cells and with BCR, CD40-L from activated B cells to stimulate B cell response during innate–adaptive immunity (95) (**Figure 6C**). The adaptive immunological response involves the generation of antibodies by plasma cells; innate immunity is regulated by IgA transcytosis in ovarian cancer (96) (**Figure 6D**). The transition from the innate immune response to adaptive immune response involves antigen presentation, followed by T cell activation, and, finally, B cell activation. The immune system is an unitary entity, and its regulation is dependent on a range of complex and diverse factors. HCs and IGLDCPs play an essential role in the three stages of the immune response, namely, innate immunity, innate–adaptive immunity, and adaptive immunity.

In the present review, we have discussed the interactive roles of HCs and IGLDCPs. Our goal is to provide novel insights based on existing concepts, and we believe that this will serve as a foundation for future research. The questions raised in the introductory section of the manuscript have been addressed and the knowledge gaps in the existing literature have been acknowledged. Along this line of investigation, potential clinical and research-related applications would greatly benefit immunological researchers and medical professionals.

## Author Contributions

K-JL, J-HM, C-XC, and JXJ structured the manuscript. K-JL, J-HM, C-XC, JXJ, HZ, and MRA wrote the manuscript. C-XC constructed the figures. K-JL, J-HM, and C-XC collected and compiled the references. K-JL, MRA, JXJ, and HZ proofread the manuscript. All authors contributed to the article and approved the submitted version.

## Funding

K-JL was supported by the National Natural Science Foundation of China (NSFC) (32160662, 31772225, 31471823, 31260448, 31060251) and the Science and Technology Planning Project in Key Areas of Yunnan Province (202001BB050002). K-JL was also supported by the Donglu Scholar Program of Yunnan University. JXJ was supported by the Welch Foundation (AQ-1507). MRA was supported by the German Federal Ministry of Education and Research (BMBF)—German Network of RASopathy Research (GeNeRARe; grant number: 01GM1902C) and the European Network on Noonan Syndrome and Related Disorders (NSEuroNet; grant number: 01GM1621B).

## Conflict of Interest

The authors declare that the research was conducted in the absence of any commercial or financial relationships that could be construed as a potential conflict of interest.

## Publisher’s Note

All claims expressed in this article are solely those of the authors and do not necessarily represent those of their affiliated organizations, or those of the publisher, the editors and the reviewers. Any product that may be evaluated in this article, or claim that may be made by its manufacturer, is not guaranteed or endorsed by the publisher.
